# From community engagement, to community-engaged research, to broadly engaged team science

**DOI:** 10.1017/cts.2017.1

**Published:** 2017-04-12

**Authors:** Harry P. Selker, Consuelo H. Wilkins

**Affiliations:** 1 President, Association for Clinical and Translational Science; 2 Chairman, Clinical Research Forum; 3 Dean, Tufts Clinical and Translational Science Institute, Tufts University; 4 Executive Director, Institute for Clinical Research and Health Policy Studies, Tufts Medical Center; 5 Executive Director, Meharry-Vanderbilt Alliance; 6 Associate Professor of Medicine, Vanderbilt University Medical Center and Meharry Medical College

## Abstract

A foundational principle and practice for translational research is active participation of a range of disciplines, referred to as “team science.” It is increasingly apparent that to be relevant and impactful, these teams must also include stakeholders outside the usual academic research community, such as patients, communities, and not-for- and for-profit organizations. To emphasize the need to link the practices of team science and of community-engaged research, we propose a framework that has community members and stakeholders as integral members of the research team, which we term, “broadly engaged team science.” Such transdisciplinary and multi-stakeholder teams will be best suited to pose translational research questions, conduct the research, and interpret and disseminate the results. We think this will generate important and impactful science, and will support the public’s regard for, and participation in, research.

## Introduction

Clinical and Translational Science Awards (CTSAs) have focused from their outset on translating research into impact on the health of the public [1]. A foundational principle of translational research is collaboration, requiring active participation of a range of disciplines. Yet, to be relevant and impactful, research teams must also include stakeholders outside the usual academic research community, including patients, communities, and not-for-profit and for-profit organizations. Although some CTSAs have successfully implemented programs in team science and community-engaged research, these efforts are often independent, leaving gaps in the translational continuum. To bridge these gaps, we propose a framework that goes beyond involving the community as advisors to a model that has community members and stakeholders as *authentic and integral members of the research team*. We term this “broadly engaged team science.”

## Objective: What Are We Trying to Achieve?

The intended objective is spawning transformative advances in the understanding of human health, medical care, and public health interventions. Beyond publishing articles that advance science as part of an academic dialog, this is about having impact via healthcare and public health measures. Even to specify such goals, we need to adopt an inclusive and participatory framework of team science that allows us to integrate diverse perspectives, develop new theories, and transcend disciplinary and role-based silos using new approaches to translational research. This means we need diverse communities who are active partners, meaningfully engaged, and committed to participating in long-term projects that hold the promise of shared benefits. This broad public engagement in medical and health research ideally should generate a shared sense of participating in research as contributing to the common good.

## Methods: How Will We Get There?

To build this effort, we must leverage and extend established methods for team science and engagement. A good exemplar is the NIH’s Precision Medicine Initiative (PMI), which will collect data from more than 1 million participants, including demographic, sociocultural, clinical, biological, sensor, behavioral, and environmental information [2]. These data will be acquired directly from participants, electronic health records, insurers, government, and other sources, and importantly they will continue to be acquired over time. PMI will leverage the many technical developments of recent years, will spur new technologies and methods, and will power extraordinary advances in healthcare and health. However, the PMI will rest on a foundation of actively engaging participant communities as partners at all levels including oversight, design, conduct, and evaluation, and that model is not completely understood or implemented. Methods must be further developed by which a broader community of scientists, academics, community members, patients, and other stakeholders come together as teams to instigate science that is innovative, rigorous, and impactful. To achieve its aim of quadruple diversity—people, geography, health status, and data types [3]—PMI will enroll participants from the general public and a range of healthcare organizations. This diverse participant group will include those traditionally underrepresented in research, such as racial and ethnic minorities, women, and those who have compromised access to healthcare. Beyond general outreach to the public and carefully conceived and executed communications, this will require authentic, deep, and durable engagement of stakeholders of all types—patient advocacy groups, faith-based organizations, community organizations, health and public service organizations, industry, governmental agencies, and others.

## Values: What Principles Will Guide Our Decisions and Actions in Getting There?

The values involved in this effort will need to be our society’s highest, and it will be incumbent to constantly exemplify them. Besides good and transparently ethical organizational practices, these will include values integral to science in general: the search for truth, integrity, challenging existing understanding, logical and evidence-based evaluation of alternatives, civility, and respect for collaborators even while critically examining ideas and data. In addition, we must prioritize trust, transparency, and an appreciation for diverse perspectives, which are critical to building high-performing teams. To meaningfully engage community stakeholders, we must value and respect all types of strengths, assets, knowledge, and experiences and develop opportunities for co-learning among team members. Not traditionally, an explicit focus of clinical research, but recently elevated by the Patient-Centered Outcomes Research Institute, are patient-centeredness and stakeholder engagement [4]. Both are deeply rooted in the needs of individuals, with authentic engagement of patients and stakeholders in generating treatments that put individual patient characteristics and preferences in the center of the work.

## Leaders: Who Will Be the Change Agents?

This effort will require leaders poised to help navigate the complex structures and hierarchy that are likely to impede such an effort. There are obstacles to bringing together research disciplines and practices. Efforts based on the principles of community engagement and focused on research in response to community priorities will need to be combined with efforts focused on removing barriers to collaboration within academic settings and the tradition of research being based on investigators’ immediate priorities. We will need to leverage the expertise among CTSAs and their long-standing commitments to community engagement and team science. Innovative programs will need to be developed within and outside CTSAs, NIH’s Institutional Development Award Clinical and Translational Research Centers, and other translational research enterprises that may help guide this new area, which also will require democratic and empowering leaders who are experienced in engaging community members and researchers across the translational spectrum.

If successful, broadly engaged team science will deliver transdisciplinary teams that carry out research in partnership with stakeholders, teams, jointly led by academia and community, will pose translational research questions, conduct the research, interpret and disseminate the results, and citizen-scientists will lead the research. Other markers of success will include increased public trust in research, more efficient research, and a more engaged, diverse research workforce. Hopefully, broadly engaged team science will not only generate better and more widely applicable research, it will catalyze a change in how the public regards science and the mutual benefit to all.
